# A High-Throughput Eight-Channel Probe Head for Murine MRI at 9.4 T

**DOI:** 10.1002/mrm.22414

**Published:** 2010-07

**Authors:** Titus Lanz, Matthias Müller, Hannah Barnes, Stefan Neubauer, Jürgen E Schneider

**Affiliations:** 1 Rapid Biomedical GmbHRimpar, Germany; 2 British Heart Foundation Experimental MR Unit, Wellcome Trust Centre for Human Genetics, Department of Cardiovascular Medicine, University of OxfordOxford, UK

**Keywords:** MRI, phased array, RF coil, parallel imaging, 9.4 T, mouse

## Abstract

Murine MRI studies are conducted on dedicated MR systems, typically equipped with ultra-high-field magnets (≥4.7 T; bore size: ∼12–25 cm), using a single transmit-receive coil (volume or surface coil in linear or quadrature mode) or a transmit-receive coil combination. Here, we report on the design and characterization of an eight-channel volume receive-coil array for murine MRI at 400 MHz. The array was combined with a volume-transmit coil and integrated into one probe head. Therefore, the animal handling is fully decoupled from the radiofrequency setup. Furthermore, fixed tune and match of the coils and a reduced number of connectors minimized the setup time. Optimized preamplifier design was essential for minimizing the noise coupling between the elements. A comprehensive characterization of transmit volume resonator and receive coil array is provided. The performance of the coil array is compared to a quadrature-driven birdcage coil with identical sensitive volume. It is shown that the miniature size of the elements resulted in coil noise domination and therefore reduced signal-to-noise-ratio performance in the center compared to the quadrature birdcage. However, it allowed for 3-fold accelerated imaging of mice in vivo, reducing scan time requirements and thus increasing the number of mice that can be scanned per unit of time. Magn Reson Med, 2010. © 2010 Wiley-Liss, Inc.

In the past decade, MRI has been well established as the most advanced noninvasive imaging modality to study numerous animal models in preclinical and basic research. The mouse in particular has emerged as the animal model of choice due to our ability to transgenically ablate, augment, or mutate specific genes and their products in this mammalian organism, which resembles human anatomy and physiology much more closely than, e.g., the zebra fish. Mice are commonly studied in dedicated MRI systems (experimental MR systems), equipped with ultra-high-field magnets (≥4.7 T) and a bore size of 12–25 cm. While the applications are not limited to a particular organ or tissue type, they are typically performed using a single transmit (Tx)-receive (Rx) coil (volume or surface coil in linear or quadrature mode) or a Tx-Rx coil combination. The concept of replacing the single (Rx) coil with an array of smaller and independent surface coils was proposed by Roemer et al. in 1990 (1). This technique is well established on clinical MR systems at magnetic field strengths of 1.5–3 T and minimal space limitations. Modern research MR systems are nowadays also fitted with more than one Rx channel in order to profit from the gain in signal-to-noise ratio (SNR) and/or from accelerated imaging, both of which are offered by coil arrays. Recent developments increased the number of receivers (and elements in the coil array) on clinical scanners up to 128 (2). This significant difference in the available number of channels between clinical and research instruments is caused by the fact that the multichannel technology at higher frequencies and smaller array geometries is technically more challenging while at the same time the market for research systems is much smaller than the clinical MR market. Two- to four-element coil arrays have been reported for different applications and field strengths (e.g., brain (3), heart (4)). Rodent applications on clinical MR systems (1.5–3 T) allowed an increase in the number of coil elements, up to 20 (5–8), while only one 16-channel volume array (i.e., 2 × 8 coils arranged cylindrically, with id of 60 mm) has been reported for rats at 7 T (9).

A second important difference between clinical whole-body and research MR systems dedicated to small animal investigations is the degree to which the scanner operation is automated. Clinical scanners are optimized for patient comfort and operator friendliness, whereas research systems are typically more flexible in order to accommodate the large variety of applications. While this flexibility is crucial for basic research, it can limit the user friendliness and sample throughput offered by these systems. This can become a major obstacle, for example, in drug or in genetic screening studies (such as large-scale mutagenesis programs), where the investigation of a high number of rodents is needed. In these cases, the emphasis of the MR system has to be on efficiency and not on flexibility. While experimental MR systems are also equipped with automated calibration routines for frequency, Tx power, or receiver gain adjustments, less attention has been paid to the hardware setup (i.e., radiofrequency [RF] coil). This includes positioning and fixation of the RF coils both inside the magnet and relative to the region of interest in the animal (potentially for each experiment), and tuning and matching, which may sometimes require ambiguous wiring and routing. This additional setup routine can be time consuming and can waste valuable (and inherently expensive) scan time. Moreover, coil arrays can add additional complexity and time to be spent on setup and positioning. The improved efficiency of clinical scanners has been achieved by using fixed tuned and matched RF coil(s), by minimizing the number of connectors through the use of multifunctional connectors, and by preinstalling the Tx coil inside the magnet, while the Rx coils are local coils that are easy to position. Thus, the operator can focus on patient care and only has to place the patient in the correct position at the beginning of the scan.

The aim of this work was to increase the number of elements in a coil array dedicated to murine MRI at an experimental ultra-high-field (9.4 T) small-bore (21 cm; gradient id: 12 cm) MR system, to transfer the setup concept for RF coils from clinical scanners to animal research, and to perform a detailed characterization of the coil array. We specifically sought to improve the workflow by optimizing the probe-head setup and to provide parallel imaging capabilities. We designed a fixed tuned and matched probe head, comprising an eight Rx element coil array in combination with a linear Tx volume resonator, which requires low setup time and is capable of accelerated MRI.

## MATERIALS AND METHODS

### General Design of the Probe Head

**FIG. 1:**
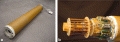
Photographic illustrations of (**a**) the entire probe head with fixation wedge at the front and the white Rx connectors at the back of the probe head, and (**b**) the combination of the birdcage resonator as the Tx coil and the volumetric eight-channel Rx array.

The standard setup for horizontal experimental MR systems is a Tx coil with a housing, which covers only a length determined by the geometric extension of the resonator itself. Specifically, making the housing as short as possible provides compactness with reduced weight. Another commonly made argument concerns the space available for the experimental setup, monitoring, and animal support. However, both arguments are not valid in most applications for the following reasons: first, Tx coils are usually placed permanently inside the magnet. Thus, compactness for easy handling is secondary. Second, the additionally available space is rarely used as this would limit the z position of the animal handling system with respect to the Tx coil. Particularly, most systems limit themselves to the inner diameter of the Tx coil over the whole length. Therefore, there is no reason why the Tx coil cannot be extended toward the end of the magnet bore. We used this argument for moving from a pure Tx coil with a short housing toward a real probe head (Fig. 1a). It can be mounted similarly to RF probes on vertical NMR systems and fixed by two screws at the magnet end and a screwable wedge at the RF coil front. Hence, the probe head can be positioned within seconds and remains stable and resistant against vibrations. The outer diameter of the probe head is 119 mm; its overall length 841 mm.

Rx arrays are typically enclosed in their own housings and mounted directly to the object under investigation. This optimizes the filling factor and minimizes the space taken up by the array. Given the small size of a mouse, the coil housings usually have approximately the same size as the animal itself. When positioning and fixing the housing to the mouse, the access for animal care may be severely restricted. Furthermore, having Tx and Rx coil(s) to position independently can make it very challenging and time consuming in order to find the correct position with respect to the mouse and the coils. We avoided this problem by including the array in the probe head itself. Therefore, the mouse can be set up without any RF coils and RF cables. The operator can fully focus on the animal and has optimal access, even when taking out the animal in between two scans. The inner diameter of the probe head is 33 mm, which was adjusted to the typical size of mouse, allowing for imaging of mice with a body weight of up to ∼30 g. It has to be noted that fixed-volume arrays usually do not optimize the filling factor, and therefore the SNR performance, as their diameter is not matched to the mouse body shape. This becomes even more pronounced if specific organs are examined. However, we accepted this drawback, as our main aim was to reduce scan time by providing parallel imaging capability.

### The Tx Coil

The Tx coil in the probe head is a high-pass birdcage resonator (10). An optimization of the filling factor for the Tx coil was not necessary as sufficient Tx power is available (nominal 1 kW). The focus was on the homogeneous excitation over the region of interest. We therefore chose the large diameter of 67 mm and a length of 82 mm. The resonator was made of printed circuit boards for the end rings and copper tubes for the 16 rungs (Fig. 1b). High-pass birdcages with a larger number of rungs have a higher frequency for the homogeneous mode when keeping the capacitances constant. This is beneficial for increasing the capacitances in order to minimize dielectric coupling to the sample and the other setup. The resonator is driven in linear mode. In order to suppress the second homogeneous mode, the end rings of the resonator were split. The coupling was achieved by a capacitive network with a serial match capacitor over two ring capacitors because the impedance of just one ring capacitor was too small to obtain an impedance of 50 Ω. In order to keep the symmetry of the resonator and to avoid cable waves, one ring capacitor was split, providing virtual ground in between the two capacitors, and a balun made of semirigid cable was used. All capacities were chosen to obtain an impedance for the resonator of 50 Ω at the working frequency of 400.0 MHz. Due to the low filling factor of the volume coil and hence the minor mismatch under physiologic loading, it is fixed tuned and matched. PIN diodes were introduced in each rung to switch off the resonator. The PIN diodes are located close to the lower end ring in order to minimize the length of the connection wires to the direct current supply. The resonator is active under current flow (100 mA per PIN diode), while each PIN diode, embedded in a resonant circuit, opens the rung under reverse voltage.

### The Rx Array

**FIG. 2:**
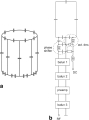
**a**: Electrical circuitry of the Rx array. The Rx array is a degenerated bandpass birdcage coil where each mesh is used as a separate coil element. **b**: Electrical circuitry of one Rx element. Tuning capacitors are distributed over the coil element. For maintaining electrical balance, the matching network is coupled to a symmetrically split tuning capacitor. A π phase shifter is used for adjusting preamplifier decoupling. Each coil element is actively switched off by a trap and a PIN diode. Baluns are used for avoiding cable waves.

The Rx array is an eight-channel volumetric array. The conductors of the array are made of flexible printed circuit board, including the coupling network. The length of the array is 33 mm, covering not the whole mouse but allowing for investigation of specific organs, such as the heart or the kidneys. We chose a shared inductor decoupling mechanism between next neighboring elements rather than decoupling by overlap in order to reduce the amount of copper within the Tx field of the resonator. Thus, the array design is close to a degenerated birdcage (11) with only eight rungs; overlap decoupling would result in 16 vertical conductors (Fig. 2a). The shared inductor design leads to an element circuitry with split capacitors (Fig. 2b), which generally minimizes electric losses in the sample but also decreases the unloaded Q. Due to the small size of the array elements and the low filling factor, coil noise is expected to dominate even at the high frequency of 400 MHz. Decoupling the elements from the end rings (in order to avoid element coupling introduced by the end-ring modes) by applying the same shared conductor method did not achieve any improvement, and the preamplifier decoupling mechanism was found to be sufficient. The coupling networks are similar to those of the Tx coil, including a split capacitor in the Rx element for achieving electrical symmetry and including baluns for ensuring full electrical balance. The array is fixed tuned and matched. This can be done without detrimental effect on the SNR as the preamplifier does not need a low reflection but noise match. The noise figure is dominated by the preamplifier's first-stage transistor. Our preamplifier uses a GaAs FET as a first stage and provides a noise figure, which is relatively robust against impedance changes. However, the design of the preamplifier is critical with respect to the noise coupling of the elements, which resulted in reengineering the preamplifiers for an optimum noise correlation of the array elements. The high impedance of the preamplifier input is not, as commonly done, transferred into a low impedance (1), but by means of a 50 Ω phase shifter into an inductance, which forms in combination with the tune and match capacitance a high-impedance trap within the coil. This preamplifier decoupling mechanism is utilized during reception for decoupling non-next-neighboring elements. The frequency of preamplifier decoupling was adjusted to the working frequency. The array elements are actively switched off by means of actively switched traps built into each element.

For user-friendly handling of the probe head, the number of connectors was reduced as far as possible. A coil combination consisting of one Tx and eight Rx channels needs at least nine RF and as many direct current lines. For ensuring compatibility with already available setups, we could reduce the connectors to a total number of four.

### Coil Characterization and MRI Experiments

The Tx coil and the Rx array were characterized on the bench, using a network analyzer (model E5061A; Agilent Technologies) and a physiologic loading phantom (aqueous solution of 100-mM NaCl filled in a cylindrical glass tube; length 59 mm, od 25 mm, equivalent to the load of a mouse). The effectiveness of active decoupling was measured by a set of two pickup loops, which are geometrically decoupled from each other.

Imaging experiments were carried out on a 9.4 T (400 MHz) MR system (Varian Inc., Palo Alto) comprising a horizontal magnet (bore size 210 mm), a VNMRS Direct DriveTM console and shielded gradient systems (600 mT/m, rise time 180 μs, id 12 cm; 1000 mT/m, rise time 130 μs, id 6 cm), and eight separate Rx channels. Comparative experiments were performed with a quadrature-driven birdcage coil of the same inner coil diameter (i.e., 33 mm) and a resonator length of 35 mm (which is a typical length for, e.g., mouse heart imaging).

The coil characterization was performed on a larger “mouse” phantom solution consisting of 20-mM NaCl + 1-mM Gd, filled in a 50-mL syringe (id 27 mm), simulating a mouse of a body weight of approximately 33 g while providing a sufficient geometrical filling of the RF coils.

The Tx efficiency was assessed using a triple spin echo sequence (echo time/pulse repetition time = 19/1000 ms; unlocalized hard pulse followed by three orthogonal slice-selective 180° refocusing pulses) to localize a 6.5 mm^3^ voxel in the isocenter. The images were phased and the signals from the voxel averaged and fitted sinusoidally to determine the length for the 360° pulse.

Homogeneity of the Tx coil of the eight-channel array was determined qualitatively by axial and coronal spin echo imaging on the syringe while driving the Tx coil in Tx-Rx mode: echo time/pulse repetition time = 8/1000 ms, 128 × 128, axial 40 × 40 mm, coronal 80 × 40 mm. The RF pulse was calibrated on a 1 mm axial slice in the isocenter. To characterize the array, images of the same sample were acquired using the volume resonator in Tx and the eight-channel array in Rx mode. Undersampled (*R* = 2, 3, and 4) data sets were generated in postprocessing followed by SENSE reconstruction. *g*-Factor maps were calculated according to (12):


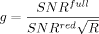
[1]

Noise data (same sequence parameters but without RF pulses) were acquired to calculate the noise correlation matrix (13) and to conduct SNR measurements, using a bootstrap method (14,15).

In vivo experiments were conducted on C57Bl/6 mice. The mice were positioned on an air-heated blanket, prone/on their left side in order to place the front limbs next to the nose cone. Fully and undersampled (*R* = 2, 3, and 4), ECG- and respiratory-gated two-dimensional spin echo-sequences (echo time = 9 ms, pulse repetition time = 1× respiratory cycle length ∼2 sec, field of view = 30 × 30 mm, slice thickness 1 mm, axial orientation, matrix size 256 × 256) were applied to mouse kidneys. The undersampled data sets were reconstructed using GRAPPA (16) (24 autocalibration lines, which were not included into the final image reconstruction).

All in vivo investigations conformed to Home Office *Guidance on the Operation of the Animals (Scientific Procedures) Act*, 1986 (HMSO) and to institutional guidelines.

## RESULTS

The Tx port showed an impedance of 50 Ω (27 dB) when loaded with the physiologic mouse phantom. There was hardly any change of the impedance when loading the Tx resonator with the phantom, due to the low filling factor. The unloaded Q of the Tx resonator measured in transmission mode (S_12_) was 300, dropping to 130 when loaded with a mouse phantom. This Q drop disappeared almost completely when introducing the Rx array, since the array screens the Tx resonator against the sample. Q-factors are not accessible in this state since the resonator is fixed tuned and matched, and the resonator will not be in a well-defined state if the impedance is not matched to 50 Ω. The Q drop of the quadrature driven birdcage coil is from 250 to 80.

The decoupling efficiency, measured as the difference between the S_12_ between two pickup loops when switching the Tx resonator on and off, was measured to be 30 dB at the working frequency (measured in various positions within the resonator), illustrating the good performance of the decoupling mechanism.

Voxel-selective measurements of the 90° pulse in the center of the Tx coil yielded a length of 102 μs at a pulse power of 650 W, resulting in a Tx efficiency of 2.3 μT/√W. The quadrature mouse birdcage resonator with the same inner diameter of 33 mm shows a Tx efficiency of 13.7 μT/√W at a 90° pulse length of 17 μs.

**FIG. 3:**
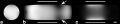
**a**: Axial and (**b**) coronal spin echo images acquired with the linear birdcage in Tx/Rx mode. **c**: Corresponding coronal view acquired with the array. Reduced sensitivity can be seen in (**b**) at the location of the array (gray lines in (**b,c**)). The arrows in (**b**) indicate a further attenuation caused by the coupling network. Scale bar: 5 mm.

Spin echo imaging on the homogeneous phantom with the Tx resonator in Tx/Rx mode shows some inhomogeneities in the outer regions (Fig. 3a). These inhomogeneities are not linked to the typical patterns of a linearly driven birdcage but result from the proximity of the Rx array. Even though the array is decoupled, the large area covered by copper affects the Tx field homogeneity. This is demonstrated by the corresponding coronal image (Fig. 3b). In the areas of the Rx array, an attenuation of the Tx amplitude of RF field can be seen. In the areas of the coupling networks, this effect is even higher. Figure 3c shows the same coronal view, but using the array in Rx mode.

The impedance of the Rx elements when loaded with the physiologic phantom ranged from −20 dB to −39 dB, with an average of −29 dB. The unloaded Q was 130, dropping to 100 when loaded with the phantom. The mean active decoupling efficiency was 32 dB, measured in the same way as for the Tx coil. The crosstalk of neighboring elements by means of shared inductor design was −19 dB, while preamplifier decoupling using the high-input reflection of the preamplifiers was used to ensure the decoupling of nonneighboring elements.

The choice of the type of preamplifiers was crucial for the performance of the array. Despite having good properties on the workbench (noise figure and input reflection), a very high noise correlation of up to 80% between the channels was observed in the experiment. This noise correlation resulted from self-oscillating properties of the preamplifiers. These can often not be detected by a simple noise figure measurement as they tend to occur at much higher frequencies than the working frequency. A further mechanism for amplifying the correlation effect is a high Q of the coil elements. A lower Q-value of the coil elements would also reduce the noise transfer between the channels (which, in our case, was observed for heavily loading phantoms). Using an appropriate preamplifier design (in this case, mainly by adjusting some ground connections to preferable positions on the PCB), we could reduce the noise correlation to the level of pure coil element coupling. The preamplifiers have a gain of 28.5 dB, a noise figure of below 1 dB, and an input reflection coefficient of S_11_ = 0.8 (magnitude; phase not recorded). This S_11_ corresponds to preamplifier decoupling of about 10 dB. The output of the preamplifiers is tuned to 50 Ω.

Table 1 shows the noise correlation matrix for (a) the mouse phantom and (b) in vivo (28.8 g mouse). The mean correlation level on the phantom was 6.4%; the maximum correlation was 16.4%, which was comparable to the in vivo results (mean 6.5%, maximum 15.7%).

**Table 1:** Noise correlation matrices for (a) mouse loading phantom and (b) mouse

**a**: Phantom (in %)							
100.0	3.6	8.4	2.1	3.0	16.4	2.5	9.9
3.6	100.0	9.5	3.6	14.5	0.1	11.4	0.6
8.4	9.5	100.0	10.2	3.3	9.5	7.2	3.7
2.1	3.6	10.2	100.0	8.3	2.8	3.6	8.8
3.0	14.5	3.3	8.3	100.0	1.3	13.3	1.9
16.4	0.1	9.5	2.8	1.3	100.0	4.5	10.7
2.5	11.4	7.2	3.6	13.3	4.5	100.0	4.8
9.9	0.6	3.7	8.8	1.9	10.7	4.8	100.0
**b**: Mouse In Vivo (28.8 g; in %)							

100.0	2.4	5.3	3.1	3.4	15.7	6.2	11.1
2.4	100.0	6.0	7.4	14.5	1.4	10.7	2.6
5.3	6.0	100.0	14.1	2.1	9.5	0.7	9.1
3.1	7.4	14.1	100.0	10.8	0.5	5.6	8.9
3.4	14.5	2.1	10.8	100.0	1.5	11.1	1.9
15.7	1.4	9.5	0.5	1.5	100.0	1.2	10.5
6.2	10.7	0.7	5.6	11.1	1.2	100.0	3.8
11.1	2.6	9.1	8.9	1.9	10.5	3.8	100.0

**FIG. 4:**
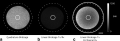
SNR-maps for (**a**) the quadrature birdcage coil, (**b**) the linear birdcage in Tx/Rx, and (**c**) the linear birdcage in Tx and the eight-channel array in Rx mode. All SNR maps were scaled equally. The mean SNR of a 5 mm circular region of interest in the center of the coil (as indicated by the white circle) was (**a**) 592, (**b**) 69, and (**c**) 271. The dashed line illustrates the inner diameter of the coils and the gray circle in (**c**), the location of the coil array. Scale bar: 5 mm.

Figure 4 shows the SNR maps for (Fig. 4a) the quadrature birdcage coil, (Fig. 4b) the linear volume resonator in Tx/Rx mode, and (Fig. 4c) the eight-channel array. The measured SNR of a 5 mm circular region of interest in the center of the coil was 592 ± 64 for the quadrature birdcage coil, 69 ± 7 for the linear Tx coil, and 271 ± 26 for the eight-channel array, respectively (mean ± standard deviation). As expected, the array outperforms the quadrature birdcage close to the array elements. However, only ∼50% of the SNR achieved with the quadrature birdcage was obtained in the center of the eight-channel array.

**FIG. 5:**
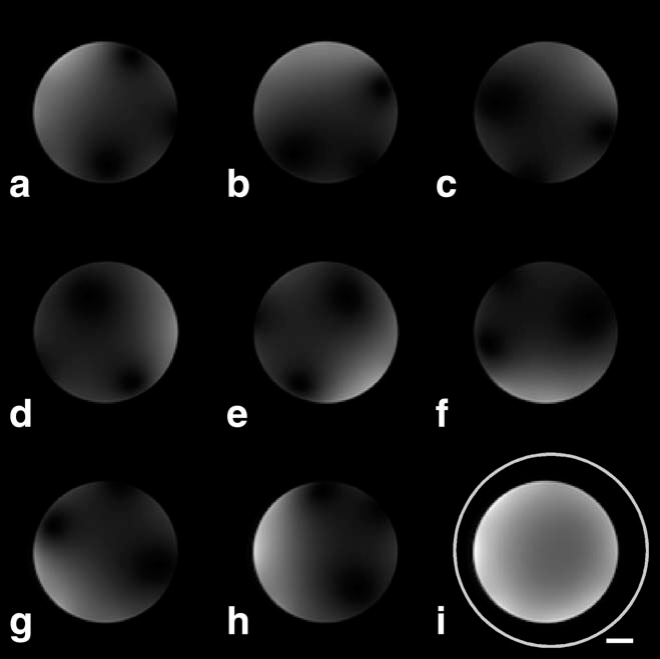
**a-h**: Individual axial coil images and (**i**) sum-of-square reconstruction of spin-echo images from the homogeneous syringe phantom (identical imaging parameters as for Fig. 4). The location of the array is indicated in (**i**). Scale bar: 5 mm.

**FIG. 6:**
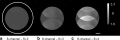
g-Factor maps for (**a**) *R* = 2, (**b**) *R* = 3, and (**c**) *R* = 4. The location of the array is indicated in (**a**). Scale bar: 5 mm.

Axial images from the individual coil elements and sum-of-square reconstruction of all coils are shown in Fig. 5. *g*-Factor maps are shown in Fig. 6, with maximum values ranging from *g*_*max*_ = 1.06 for *R* = 2, *g*_*max*_ = 1.32 for *R* = 3, and *g*_*max*_ = 2.25 for *R* = 4, respectively. These results indicate that acceleration factors of 3 will be feasible in vivo.

**FIG. 7:**
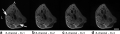
Renal imaging of a mouse using a spin echo sequence with undersampling factors of (**a**) *R* = 1, (**b**) *R* = 2, (**c**) *R* = 3, and (**d**) *R* = 4, respectively, followed by GRAPPA reconstruction. The arrows indicate the left and right kidney. (A, anterior; L, left; scale bar – 5 mm.)

This was confirmed by the in vivo application as demonstrated in Fig. 7, where axial slices through the lower abdomen of a mouse obtained with acceleration factors *R* = 1 up to *R* = 4 are shown. Twenty-four autocalibration lines were used for the GRAPPA reconstruction but not incorporated into the final data set. Figure 7c proves that a 3-fold reduction in scan time is achievable with sufficient image quality.

Setup time for the eight-channel array was approximately 1 min. Importantly, the probe-head positioning approach with only two screws for fixation at a well-defined position, the fixed tune and match approach, and the low number of connectors saved up to 10 min in the preparation of the MR scan.

## DISCUSSION

We have introduced a fixed tuned and matched probe head, dedicated to murine MRI at 9.4 T, consisting of eight Rx elements combined with a linear Tx volume resonator. Tx coil and Rx array were comprehensively characterized and were compared to a quadrature-driven birdcage coil with the same available space for the sample.

The Tx efficiency on the workbench, as well as in the NMR system, is low compared to a quadrature birdcage with the equivalent sample volume. This is mainly due to the enlarged diameter of the Tx resonator and, in addition, due to the lack of quadrature polarization. Nevertheless, the available nominal Tx power of 1 kW is sufficient for excitation in fast gradient echo imaging (as used for scouting in vivo; data not shown) or in spin echo imaging.

Tx homogeneity in the transverse plane shows significant influence of the presence of the Rx array on the amplitude of RF field of the Tx coil. Due to the huge difference in geometrical size between Tx coil and sample, one would expect a highly homogeneous amplitude of RF field pattern. However, the conducting components of the Rx array in close proximity to the sample screen the sample against the amplitude of RF field of the Tx coil. This effect is more pronounced at the level of the coupling electronics where in the center a drop-off of ∼50% of the Tx field exists compared to regions outside the Rx array. This shielding effect is due to the massive presence of conductors and electrical components, covering 17% of the surface in the area of the array and 43% in the area of the coupling networks. Reducing this covered area by smaller components or conductors with a decreased width would be possible. However, the Tx homogeneity is not impaired within the VOI, and therefore a change of the design is not necessary.

Sufficient decoupling of the Rx elements has been demonstrated on the workbench by characterizing S_12_ between neighboring channels and preamplifier input S_11_, as well as in the MR system by individual channel performance and noise correlation measurements. For S_12_ between neighboring elements, a very good decoupling of 19 dB was achieved, with a further improvement of 10 dB by the preamplifier decoupling. This degree of preamplifier decoupling was sufficient for all other pairs of array elements. Reconstructing the images from the individual channels shows that this is also obtained in the MR experiment (Fig. 5). Furthermore, the noise correlation of all channels was low, with a maximal value of 17% (Table 1).

The Q drop from an unloaded to loaded state indicates that the Rx sensitivity is not optimal and the coil noise dominates due to the small element size. This is amplified further by the volumetric coil design, which leads to a limited filling factor on a mouse (for an optimized filling factor, a fully cylindrical mouse with an outer diameter of 33 mm would be needed). While ideally the SNR in the center of an optimal volumetric array is expected to be the same as for a quadrature birdcage with a matching sensitive volume, a reduced SNR (approximately 50%) was observed in our case. However, in the outer regions, the array by far outperforms the birdcage (>factor 2). These observations suggest that the limit of SNR gain by array technology is reached for mouse applications in volumetric designs, even at high frequencies such as 400 MHz (see also Kumar et al. (17)). Improvements are certainly possible by minimizing coil losses by using wires instead of printed circuit boards or by cooling the coil. Nevertheless, from a practical point of view a carefully designed four-element volumetric array might currently be the practical limit for SNR optimized volumetric array technology in mice.

Importantly, the array provides parallel imaging capability. Acceleration properties are similar to those known from eight-element coils for human applications. Here, 3-fold accelerations are typically feasible for eight-channel head coils before excessive *g*-factors occur. The corresponding maximum *g*-factor of 1.3 observed in our study is in the range where reduced image quality due to accelerated acquisition is commonly still accepted.

The gain in SNR in the outer regions, combined with parallel imaging, should particularly benefit applications where the area of interest can be placed close to the array, including renal MRI (as demonstrated here), cardiac applications, or oncology, where frequently superficial tumors are induced. Future work is needed to evaluate the benefit of our design for these applications.

Another important goal of the probe-head design was to minimize the setup time. The fixed tuning and matching of all nine channels (i.e., Tx and Rx), the easy and rapid positioning and fixation mechanism without any adjustments, and the low number of connections require a setup time of about 1 min only. Since the animal preparation is totally independent of the RF setup, the entire RF side can be prepared *prior* to anesthetizing a mouse, reducing the period of anesthesia by ∼5–10 min. Therefore, this gain in time can either be used for additional scans without extending the total procedure time or for reducing the anesthetic burden, allowing for investigating mouse models that are sensitive to anesthesia and to increase throughput. Furthermore, when taking out the mouse in between two scans (for example, for drug or contrast injection), this setup proves very valuable and time saving. Multicoil/multimouse MRI has been reported as an alternative high-throughput approach (18,19). However, shimming, physiologic gating, and animal monitoring represent technical challenges, which may at present limit the range of applicable MR techniques.

## CONCLUSION

In conclusion, this is the first report on the design and characterization of an eight-channel Rx-coil array for murine MRI at 400 MHz. The array, which was combined with a volume-Tx coil, was integrated into one probe head. Decoupling the animal handling fully from the RF setup, providing fixed tune and match of the coils, and reducing the number of connectors minimized the setup time of the coil. Optimized preamplifier design was essential for minimizing the noise coupling between the elements. The miniature size of the elements resulted in coil noise domination and therefore reduced SNR performance in the center compared to a matching quadrature birdcage. However, it allowed for 3-fold accelerated imaging of mouse kidneys in vivo, reducing scan time requirements and potentially increasing the number of mice that can be scanned per unit of time.
